# Paclitaxel-loaded phosphonated calixarene nanovesicles as a modular drug delivery platform

**DOI:** 10.1038/srep23489

**Published:** 2016-03-24

**Authors:** Jingxin Mo, Paul K. Eggers, Zhi-xiang Yuan, Colin L. Raston, Lee Yong Lim

**Affiliations:** 1 Pharmacy, School of Medicine and Pharmacology, The University of Western Australia, 35 Stirling Highway, Crawley, WA, 6009, Australia; 2 Key Laboratory for Stem Cells and Tissue Engineering (Sun Yat-sen University), Ministry of Education, Guangzhou, Guangdong, 510080, China; 3 School of Chemistry and Biochemistry, The University of Western Australia, 35 Stirling Highway, Crawley, WA, 6009, Australia; 4 Department of Pharmacy, College of Veterinary Medicine, Sichuan Agricultural University, Chengdu, Sichuan, 611130, China; 5 Centre for NanoScale Science and Technology, School of Chemical and Physical Sciences, Flinders University, Bedford Park, SA, 5042, Australia

## Abstract

A modular *p*-phosphonated calix[4]arene vesicle (PCV) loaded with paclitaxel (PTX) and conjugated with folic acid as a cancer targeting ligand has been prepared using a thin film-sonication method. It has a pH-responsive capacity to trigger the release of the encapsulated PTX payload under mildly acidic conditions. PTX-loaded PCV conjugated with alkyne-modified PEG-folic acid ligands prepared via click ligation (fP-PCV_PTX_) has enhanced potency against folate receptor (FR)-positive SKOV-3 ovarian tumour cells over FR-negative A549 lung tumour cells. Moreover, fP-PCV_PTX_ is also four times more potent than the non-targeting PCV_PTX_ platform towards SKOV-3 cells. Overall, as a delivery platform the PCVs have the potential to enhance efficacy of anticancer drugs by targeting a chemotherapeutic payload specifically to tumours and triggering the release of the encapsulated drug in the vicinity of cancer cells.

The ability to direct a drug payload specifically and solely to a particular cellular type is regarded as a Holy Grail in chemotherapy. Most current chemotherapeutic agents are highly efficacious, but they are also associated with debilitating toxicity, essentially due to their indiscriminate attack on both cancerous and rapidly dividing healthy cells. To improve treatment outcomes, several targeting drug delivery approaches have been devised with various degrees of success. One approach is to design pH-sensitive polymeric platforms to trigger the release of a drug payload upon extravasation into cancer tissues, the optimal system being capable of responding with adequate discrimination to the small pH difference between blood and tumour milieu (pH 7.4 *vs.* pH 5.5)^1^,^2^,^3^. Another approach is to target receptors that are overly expressed on cancer cell membranes, in particular the folate receptor (FR) which has been the target of choice for a diverse range of delivery platforms, including pegylated micelles^4^,^5^,^6^, vesicles modified with acid-triggered drug releasing mechanisms^7^ and derivatised liposomes^8^. The basis for this approach stems from the essential role that folic acid (FA) plays in cell division and DNA synthesis, and the significant upregulation of FR in a number of epithelial cancers, e.g. ovarian tumours^9^, to meet the increased demand for FA during cell proliferation. Binding of FA to the membrane FR initiates FA internalization as well as any drug delivery platform strategically conjugated to the FA. Subsequent sequestration of the contents into acidic endosomes ensures that the approach does not only result in cancer-directed drug delivery, but has the potential to also enhance cellular uptake of the delivered drug. This coupled with the low to absent FR expression on normal cells^10^, and FA being a relatively cheap and commercially available small molecule that is amendable to chemical modification without losing its FR binding efficiency^11^, has made the FR-targeting approach a popular route for directing chemotherapeutics into tumours^12^.

This paper describes an extension of our work on calix[4]arene-based phospholipid mimic vesicles (PCV) by incorporating the above two cancer targeting strategies to direct the delivery of the potent but toxic drug, paclitaxel (PTX), to ovarian cancer cells. We have previously shown such calix[4]arenes are non-toxic^13^ and potent anti-oxidants^14^ with innate capacity to assemble into micelles and vesicles in aqueous media. The resultant vesicles can be wrapped with polymers for increased stability^15^, tagged with fluorescent molecules for tracking^15^, intercalated with non-polar drugs^14^, as well as hosting a polar carboplatin molecule in its molecular cavity^16^. In the present work we demonstrate a facile click-based method to modularly functionalize the PCV surface with long chain polyethylene glycol (PEG) and folic acid to provide a targeted delivery system for PTX. PTX is a widely used potent chemotherapeutic agent with intractable water insolubility and significant dose-limiting cardiotoxicity^17^,^18^,^19^,^20^. A liposomal PTX formulation has been developed to resolve the solubility issues and has recently become available to clinicians^21^. Compared to the cremophor-based paclitaxel formulation, this novel formulation has a better safety profile^21^. However, this formulation has no built-in active targeting capacity and thus exhibits similar antitumor effects^22^. We are endeavouring to develop a robust delivery platform that can be readily modified to incorporate targeting and pH-triggered drug release capabilities to improve the therapeutic index of PTX, and by extension, any other potent chemotherapeutic payload. Indeed, as described herein, the proof-of-concept PTX-loaded folate-PEG PCV exhibits significantly enhanced drug efficacy and discriminated with high selectivity towards cell lines with increased FR expression.

## Results and Discussion

### Design and Synthesis of the Calixarenes

**Figure 1:**
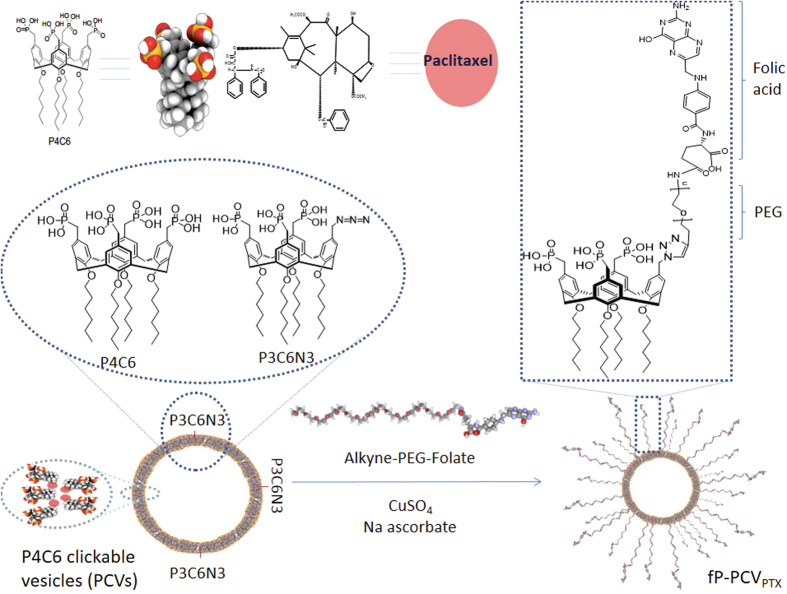
Click-based modular *drop-in* strategy for the preparation of PTX-loaded, folate-PEG P4C6 vesicles (fP-PCV_PTX_).

The calix[4]arene based phospholipid mimic described herein (Fig. 1) is amphiphilic with polar phosphonate/phosphonic acid “head” groups and non-polar aliphatic “tails”. This structure mimics phospholipids which make up the membrane of animal cells with the exception that the calix[4]arene forms a rigid “cup” or scaffold where the four head groups are constrained to point in one direction, with the alkyl chain “tails” in the other. This construct lends itself to be able to replace one of the four phosphonate head groups in the molecule P4C6 with an azide “linker” group as the molecule P3C6N3, without significantly disrupting the structural stability of the ensuing self assembled vesicles when P3C6N3 is mixed with P4C6. The azide linker enables modification of the surface of the vesicles with targeting ligands and/or imaging moieties through “Click” chemistry. Such chemistry can be performed under biologically relevant conditions, and has fast kinetics with high chemo- and regio-specificity, forming a single reaction product in high yield^23^. We have found that tethering the targeting ligands to the vesicle surface can be achieved under mild reaction conditions within the vesicle forming media which is deemed necessary to preserve both the integrity of the sensitive targeting molecules^24^ and the stability of the vesicles.

The synthesis of P4C6 has been described previously^16^ and the synthesis of P3C6N3 was accomplished *via* modifying a method described for the synthesis of the corresponding *n*-dodecyl substituted calixarenes, rather than the *n*-hexyl in the present study^15^. As described in detail in the ESI, this included standard calix[4]arene synthesis followed by addition of the *n*-hexyl groups through bromohexane and sodium hydride in DMF, formylation via the Duff reaction, reduction to the alcohol with sodium borohydride, chlorination with thionyl chloride, phosphorylation with triethylphosphite in a three to one calix[4]arene ratio, deprotection using bromotrimethylsilane followed by reaction with sodium azide to form the final compound, P3C6N3.

### Fabrication of the Phosphonated Calix[4]arene Vesicles

Phosphonated calix[4]arene vesicles (PCV) were prepared by mixing P4C6 with P3C6N3 (1 mol % relative to P4C6) using the thin-film method^25^. The hydrodynamic diameter (*D*_H_) of the PCVs was 112 ± 8 nm as measured by dynamic light scattering, and the zeta potential (ζ) was −38.76 ± 3.94 mV at pH 7.0, 25 °C, indicating a high degree of stability in aqueous media.

### Paclitaxel Loading and Release

**Figure 2:**
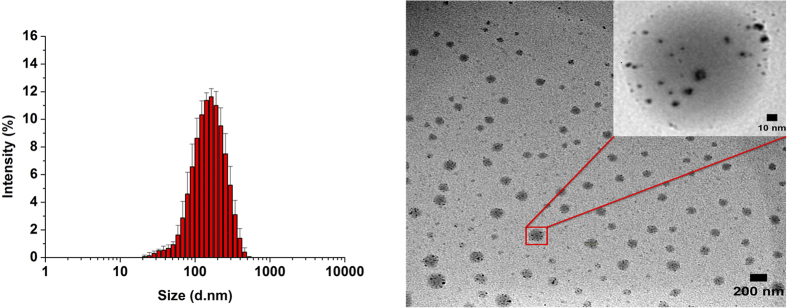
PTX-loaded *p*-phosphonated calix[4]arene vesicles (PCV_PTX_): (**A**) DLS of PCV_PTX_, Z-average size is 139 ± 16 nm, and (**B**) TEM image, scale bar 200 nm (10 nm for inset).

PTX-loaded phosphonated calix[4]arene vesicles (PCV_PTX_) were prepared using the thin-film method^25^ with a PCV:PTX molar ratio of 4:1. Loading efficiency and encapsulation efficiency of PTX were 4.03 ± 0.42% and 90.21 ± 4.84% (W/W, n = 3), respectively. The PCV_PTX_ had *D*_H_ = 139 ± 16 nm, which was larger than the blank PCV, and ζ = −40.8 ± 5.87 mV at pH 7.0, 25 °C (Fig. 2), indicating that the PTX loading did not disrupt the PCV stability.

**Figure 3:**
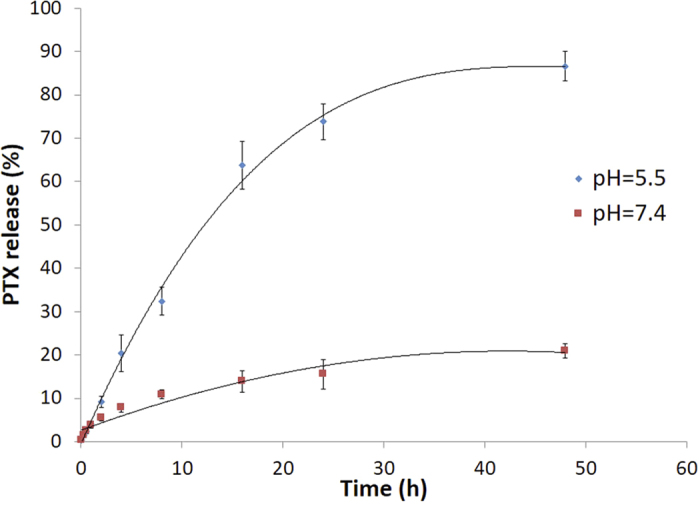
Time-dependent PTX-release profiles of PTX-loaded *p*-phosphonated calix[4]arene vesicles (PCV_PTX_) at 37 °C, into pH 5.5 (diamond symbol) and pH 7.4 (square) phosphate buffer media.

The drug release kinetics for PCV_PTX_ (Fig. 3) were monitored at both normal tissue pH (pH 7.4^26^) and cancer tissue pH (pH 5.5^26^) using the previously described LC-TOF/MS assay method for PTX^25^. As anticipated from zeta potential measurements, PCV_PTX_ demonstrated a degree of stability at the normal tissue pH of 7.4, with only 20% of the PTX load released after 48 h at 37 °C. However, at the cancer tissue pH of 5.5 at 37 °C, 75% of the PTX load was released after 24 h, and 85% after 48 h. As eluded to previously, the enhanced drug releasing capacity of the PCV platform under acidic conditions compared to slightly basic conditions appears to be related to the first pK_a_ of 7.2 for the phosphonic acid head groups^27^. At pH 7.4 the majority of phosphonic acid-head groups are expected to be singly deprotonated with a negative charge, whereas at pH 5.5, the majority of head groups are expected to be fully protonated, with the neutral molecules having significantly greater hydrophobic character. The loss of charge and the accompanying hydrophobic character implies that the vesicles will be destabilized within an acidic aqueous environment relative to the mildly basic pH 7.4 medium. This is important for chemotherapy as the vesicles will have a greater stability in systemic circulation at pH 7.4, with potential for complete PTX release following extravasation and retention in tumorigenic tissues where the pH in the extracellular milieu and in cytoplasmic lysosomes can decrease to pH 5.5.

### Tethering of Folic Acid to the Vesicle Surface

As discussed in the introduction, folic acid (FA) was chosen as the cancer targeting moiety. Poly (ethylene glycol) was incorporated as a spacer between the FA and the vesicle surface to enhance aqueous solubility, optimise the targeting activity of the surface-attached FA, and increase the potential for prolongation of systemic half-life of the vesicle, thus allowing for multiple passes of the vesicle through the targeted tumour site^28^. As the level of P3C6N3-PEG-Folate incorporated into the PCVs was relatively low (0.73% of P4C6), the folate acid would not affect the paclitaxel release profile of fP-PCV_PTX_, and this has been confirmed by comparing the paclitaxel release profile of fP-PCV_PTX_ to that of PCV_PTX_.

**Figure 4:**
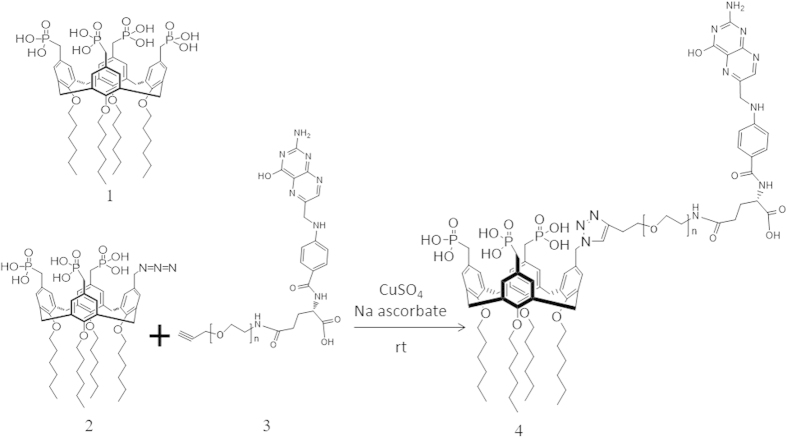
Model ‘click’ reaction between the alkyne-PEG-folate reagent and P3C6N3 in the calixarene vesicle. **1.** P4C6, **2.** P3C6N3, **3.** Alkyne-PEG-folate, **4.** P3C6N3-PEG-folate.

**Figure 5:**
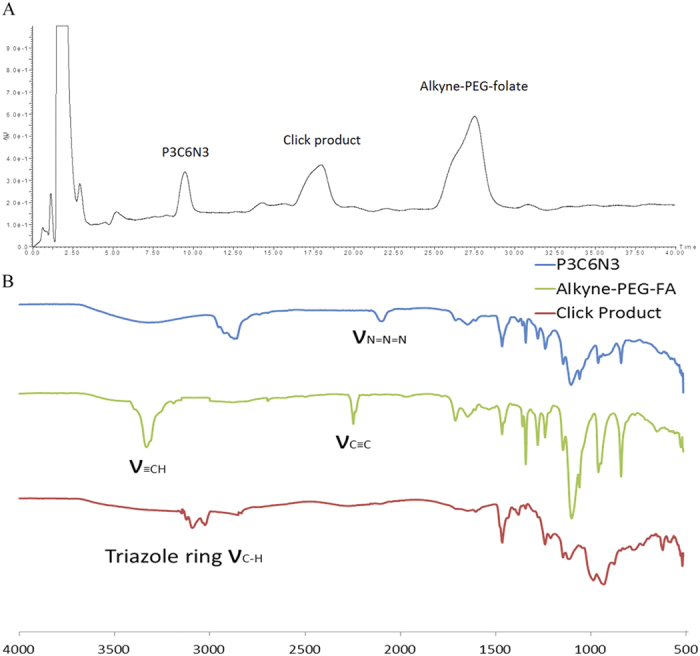
(**A**) Reverse-phase HPLC chromatogram of the crude click chemistry reaction mixture. (**B**) The FTIR spectra of the P3C6N3, Alkyne-PEG-Folate, and the click product. Peaks for azido and alkyne moieties were not apparent in the spectrum for the “click” product.

As shown in Fig. 1, the FA residue was incorporated by attaching an alkyne-PEG-folate reagent to the azide-functionalized PCV_PTX_ using a ‘Click’ reaction. To establish the conditions for the click reaction, equimolar amounts of alkyne-PEG-folate reagent and P3C6N3 (azide-functionality incorporated in PCV_PTX_) were mixed in the presence of CuSO_4_ and sodium ascorbate (Fig. 4). FTIR, Fig. 5B, establishes that both the P3C6N3 azide absorption at 2122 cm^−1^ and the alkyne-PEG-folate alkyne absorption at 3333 cm^−1^ largely disappear, with the appearance of an absorption band at 3136 cm^−1^ which corresponds to a triazole. This establishes that the PEG-folate is tethered to calixarene molecules within the vesicle. It was not anticipated that the reaction would go beyond 50% as vesicles have an inner surface and an outer surface, and the mild conditions of the click reaction were expected to only modify the outer surface. Scrambling of calixarene molecules between the outer and inner layers of the vesicle bilayer is expected to require mechanical energy input, as in the use of the thin film vortex fluidic device for vesicles built of P4C8^16^. However, HPLC of the deconstructed vesicle, Fig. 5A, established a 75% conversion of available triazide sites. The reaction was then repeated by treating the parent azide-functionalized PCV_PTX_ with a lower 1 mol % (relative to P4C6 in the PCVs) of the alkyne-PEG-folate reagent, under similar conditions. The level of P3C6N3-PEG-Folate incorporated on the PCVs was kept below 1 mol % of P4C6, noting that ~0.5 mol % of folate per liposome is reported as an optimized value for targeting studies^29^. After the “click” reaction and purification, the resulting folate-modified PCVs (fP-PCVs) contained approximately 0.73 mol % of P3C6N3-PEG-Folate, as determined by UV–vis spectroscopy (Fig. S1 in Supporting Information).

### Cellular Uptake of fP-PCV_FITC_

**Figure 6:**
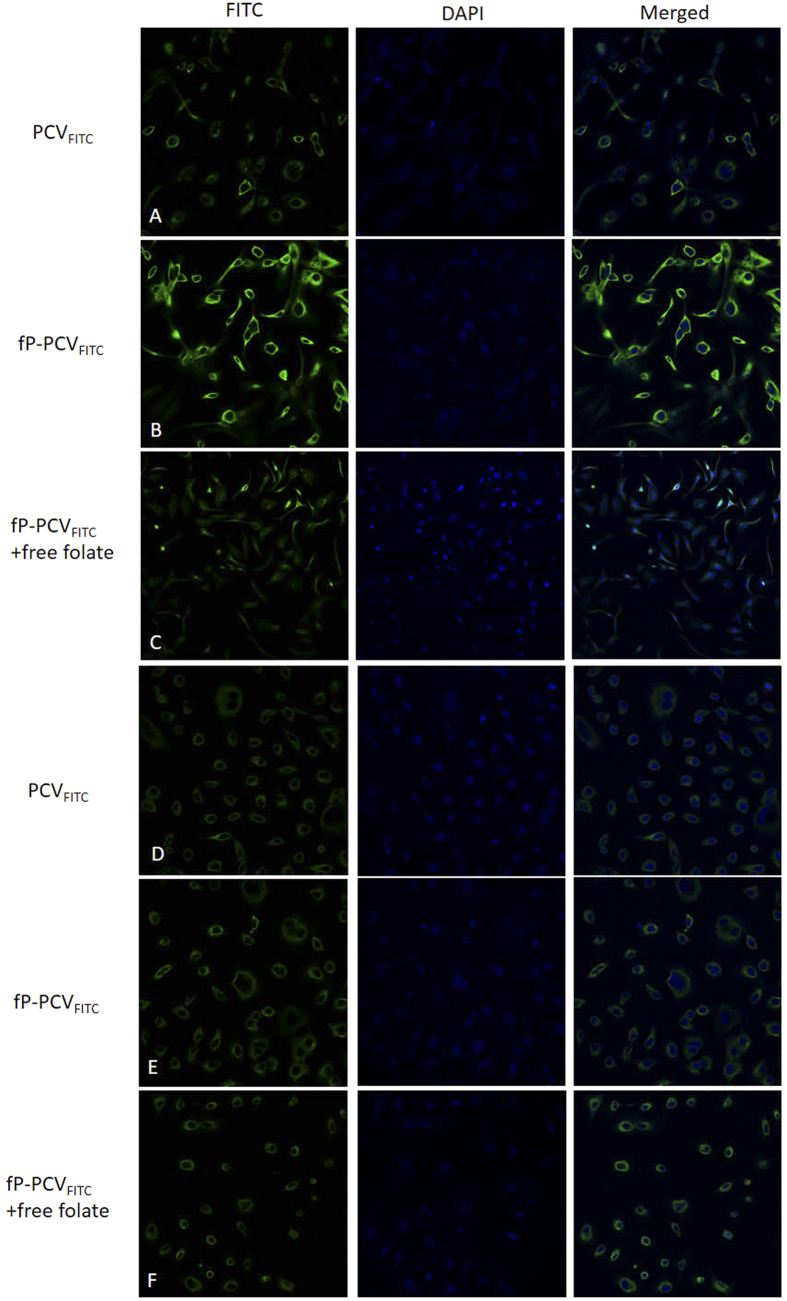
Confocal laser-scanning fluorescence microscopy images of SKOV-3 cells (**A–C**) and A549 cells (**D–F**) after 8 h incubation with PCV_FITC_ (**A,D**), fP-PCV_FITC_ (**B,E**), and fP-PCV_FITC_ with 2 mM folate (**C,F**), and then dying with DAPI.

As described in the introduction, folate receptor (FR) mediated endocytosis has been shown to also mediate the cellular uptake of moieties attached to the folate molecule. Here we provide evidence that the attached folate assists fP-PCV_FITC_ to undergo endocytosis by exposing FR-overexpressing SKOV-3 human ovarian carcinoma cells and FR-negative A549 human epithelial lung carcinoma cells to either PCV_FITC_, fP-PCV_FITC_ or fP-PCV_FITC_ with excess free folate. The CLSM images of all the incubated cells are shown in Fig. 6.

As shown in Fig. 6, the greatest FITC fluorescence was detected in the SKOV-3 cells exposed to fP-PCV_FITC_ alone (Fig. 6B) compared to those incubated with PCV_FITC_ (Fig. 6A), fP-PCV_FITC_ with free folate (Fig. 6C) or any of the exposed A549 cells (Fig. 6D–F). The variation of intensity of fluorescent signals shown between the fluorescence experiments of Fig. 6 agrees with the expectation that folate assisted FR-mediated endocytosis will provide the mechanism to assist in the uptake of folate tagged fP-PCV_FITC_. For instance, Fig. 6A shows SKOV-3 cells exposed to PCV_FITC_ which lacks the folate tag and Fig. 6B shows SKOV-3 cells exposed to fP-PCV_FITC_, with the sharp decrease in fluorescence between Fig. 6A,B strongly indicates that the folate tag on the PCVs plays a significant role in the uptake of the FITC payload by the cells. This is further emphasised by the sharp decrease in fluorescence when a significant excess of free folate is added to the media prior to fP-PCV_FITC_ being incubated with SKOV-3 cells (Fig. 6C). In this experiment the free folate binds to the FR of the SKOV-3 cells, and hence blocks the fP-PCV_FITC_ from binding to the receptors. The significant drop in fluorescence gives further evidence that it is attachment of the folate to the PCVs that assists in the endocytosis of the PCVs.

As a control, A549 cells which lack the FR were incubated with the same samples as the SKOV-3 cells and in all cases the A549 cells showed very low fluorescence of approximately the same intensity. This indicates a similar but very low uptake of the PCVs, thereby providing further evidence that both the folate receptor and the folate tag on fP-PCV_FITC_ are responsible for the strong fluorescence in Fig. 6B. This in turn supports our hypothesis that the internalization of fP-PCV_FITC_ by the SKOV-3 cells is mediated by the folate receptor.

**Figure 7:**
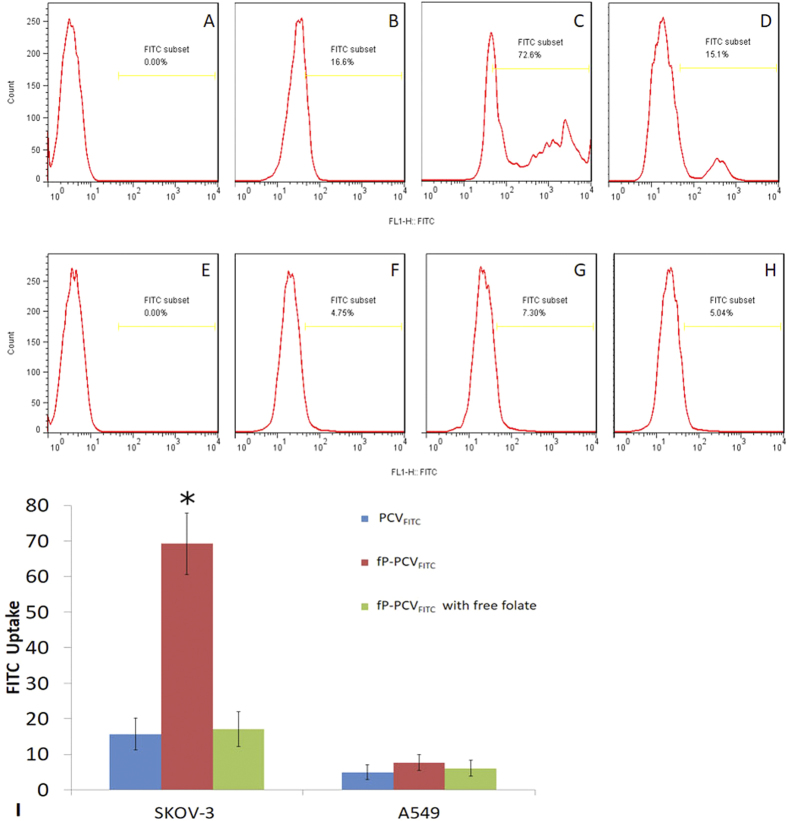
FITC intensity analysed by flow cytometry of SKOV-3 cells (**A–D**) and A549 cells (**E–H**) after 8 h incubation with cell culture media (**A,E**), PCV_FITC_ (**B,F**), fP-PCV_FITC_ (**C,G**), and fP-PCV_FITC_ with 2 mM folate (**D,H**). Exposure to PCV_FITC_ (**B**) and fP-PCV_FITC_ (**C**) led to 16.6% and 72.6%, respectively, of SKOV-3 cells to be located in the FITC subset, indicating a 337% increase in active cellular internalisation of the vesicles tagged with the targeting ligand. (**I**) Bar graph showing FITC uptake from various FITC-loaded vesicle formulations by the SKOV-3 and A549 cells. Statistical analysis was performed using One-way ANOVA test. Values of *P < 0.05 was considered statistically significant.

The incubated cells used in the fluorescence study described above were also analysed by flow cytometry. The gate of FITC was established using control groups of SKOV-3 cells and A549 cells, as shown in Fig. 7A,E. The quantitative flow cytometry data shown in Fig. 7 is consistent with the CLSM images of fluorescence in Fig. 6, indicating that fP-PCV_FITC_ is actively internalized by SKOV-3 cells while PCV_FITC_ is entering the SKOV-3 and A549 cells by non-specific adsorptive endocytosis. For instance, 16.6% of SKOV-3 cells are located in the FITC subset when incubated with PCV_FITC_ (Fig. 7B) in comparison to 72.6% when incubated with the folate tagged fP-PCV_FITC_ (Fig. 7C). This establishes a 337% increase in uptake when the targeting tag is used. Furthermore, the shape of the FITC intensity graph in Fig. 7B shows a gradual decrease in PCV_FITC_ incubated cell numbers going into the FITC subset which implies a passive process of internalization, while the multi-peaks within the FITC subset of the fP-PCV_FITC_ incubated cells (Fig. 7C) indicate process(es) other than passive permeation through the cell membrane. When the folate receptors were blocked by excess free folate (Fig. 7D), the cellular uptake of fP-PCV_FITC_ dropped to 15.1%, which is similar to the level of untagged PCV_FITC_. This would appear to indicate that the passive permeation of the SKOV-3 cell membrane by both fP-PCV_FITC_ and PCV_FITC_ is around 15%, however, the small multipeak shown in Fig. 7D suggests that some of the fP-PCV_FITC_ is internalised through some multivalent FR binding sites which are not accessible to the free folate. The flow cytometry FITC subset data for the A549 cells incubated with either PCV_FITC_, fP-PCV_FITC_ or fP-PCV_FITC_ including excess folate were between 5–7% (Fig. 7F–H) and had a steady decrease of cell numbers in the FTIC subset. This indicates that only passive permeation of the cell membrane occurs without the folate receptors on the surface of the A549 cells.

### PCV - Paclitaxel Efficacy

As mentioned in the introduction, PTX is an effective anti-cancer drug, however, it also kills normal cells that reproduce rapidly which results in serious side effects. A PTX delivery system that releases the drug primarily to cancer cells would lower side effects and improve the therapeutic outcomes. The fluorescent studies have shown that the folate tagged fP-PCV_FITC_ increases the uptake of an intercalated dye by 337% over the untagged PCV, while the *in vitro* dissolution studies have shown that in aqueous media with a similar pH to the cancer milieu, there is a higher rate of release of PTX than in aqueous media with a similar pH to blood. Next, we investigate how the folate tagged fP-PCV_PTX_ affects the efficacy of intercalated PTX through *in vitro* cytotoxicity experiments.

**Figure 8:**
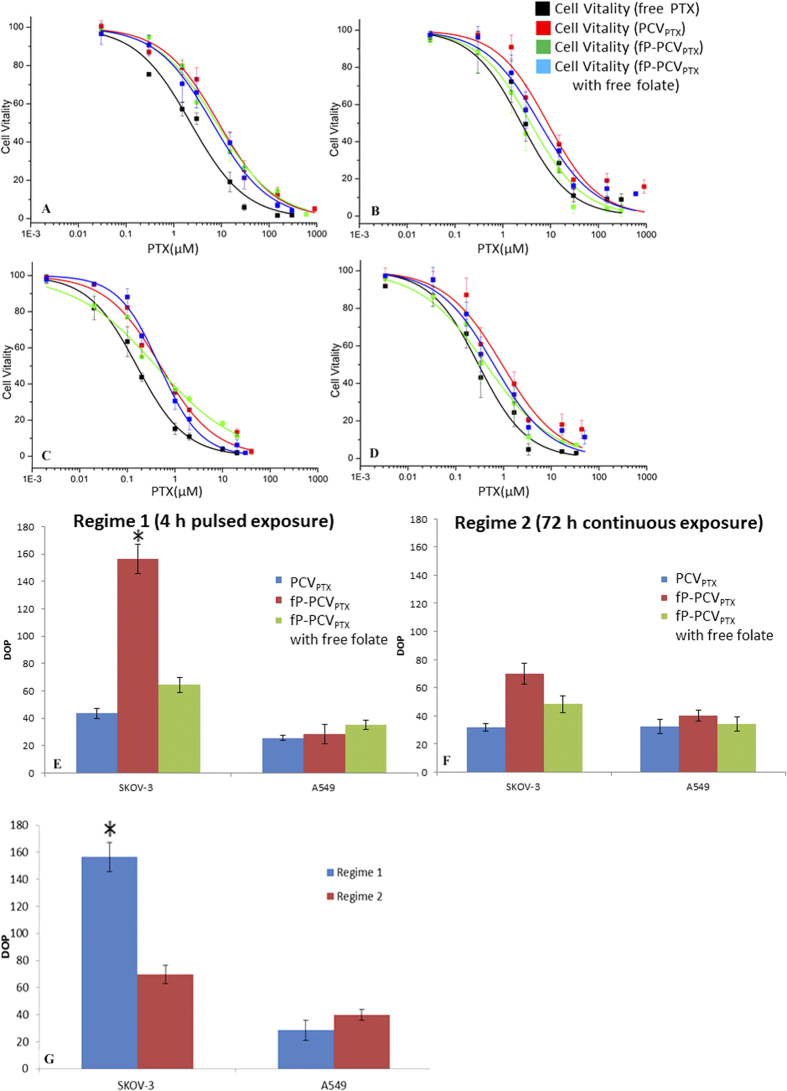
(**A–F**) Cytotoxicity profiles for various PTX formulations against FR-overexpressing SKOV-3 cells and FR-negative A549 cells. Short incubation regime 1: (**A**) A549, (**B**) SKOV-3 cells that were exposed to PTX formulations for 4 h followed by 68 h post-incubation in drug-free media. Long incubation regime 2: (**C**) A549, (**D**) SKOV-3 cells that were incubated with PTX formulations for 72 h. The PTX formulations contained free paclitaxel (PTX), PTX-encapsulated phosphonated calix[4]arene vesicles (PCV_PTX_), and PTX-encapsulated, folate-conjugated PCV (fP-PCV_PTX_) with or without free folate. Relative degrees of potentiation (DOP) of various PTX-loaded vesicle formulations compared to free PTX, calculated as [IC_50_(free drug)/IC_50_(drug in carrier) ×100] for each cell line following the pulsed (**E**) and continuous (**F**) incubation regime. (**G**) Bar graph showing the DOP of fP-PCV_PTX_ for each cell type in short and long incubation regimes. Statistical analysis was performed using One-way ANOVA test. Values of *P < 0.05 was considered statistically significant.

The FR-positive SKOV-3 cells and FR-deficient A549 cells were exposed to PTX presented as either free PTX, PCV_PTX_, fP-PCV_PTX_ or fP-PCV_PTX_ with free folate. The incubation regimes involved either a pulsed exposure, where the cells were incubated with PTX for 4 h followed by 68 h incubation in drug-free media, or a continuous exposure, where the cells were incubated for 72 h in drug-containing media. Post-exposure, the number of viable cells was counted by flow cytometry and expressed as a percentage relative to drug-naïve control cells. From the cell viability-PTX concentration plots in Fig. 8A–D, the half-maximum inhibitory concentrations (IC_50_ values) were calculated and are shown in Table 1. The degrees of potentiation (DOP)^30^ were calculated using equation 1 and are shown in Fig. 8E–G.


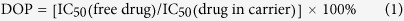


The DOP in Fig. 8E–G clearly show the relative potency of PCV_PTX_ and fP-PCV_PTX_ compared to free PTX. However, what is important for reducing the side effects is the difference in DOP between the targeted fP-PCV_PTX_ and non-targeted PCV_PTX_. In SKOV-3 cells, short exposure to fP-PCV_PTX_ (DOP = 157%) is 3.6 fold more likely to result in cell death than short exposure to PCV_PTX_ (DOP = 44%), indicating the higher potency of PTX when it is delivered *via* a targeting delivery platform. The capacity of fP-PCV_PTX_ to be specific in its delivery of the drug payload to the targeted cells can be evaluated by comparing the responses of the different cell types. For instance, DOP values show the fP-PCV_PTX_ to be 5.5 times more likely to kill the SKOV-3 cells to which it is targeted than the A549 cells. Conversely, non-specific adsorptive endocytosis is the dominant mechanism for PCV_PTX_ uptake as the FR-negative A549 cells show no measurable difference in cell viability responses to the two formulations.

As expected from the fluorescence study, the cytotoxicity of fP-PCV_PTX_ against the SKOV-3 cells was reduced about two-fold in the presence of free folate (Table 1), but the competing folate was unable to completely block the enhanced cytotoxicity conferred by the targeting fP-PCV_PTX_ over the non-targeting PCV_PTX_, as indicated by the different IC_50_ values. This enhanced potency of fP-PCV_PTX_ against the SKOV-3 cells may result from multivalent FR binding sites which are not accessible to the free folate^31^. Furthermore, this was not due to the cytotoxicity of the P4C6 as a 76-fold higher concentration of P4C6 alone (78 μM)^16^ was required to yield the same cytotoxic effect as PCV_PTX_ against the SKOV-3 cells over 72 h.

Long incubation regimes highlight the reduction in difference of cytotoxicities between fP-PCV_PTX_ and PCV_PTX_ toward SKOV-3 cells due to sufficient internalization of non-targeted PCV_PTX_ over the longer period (Fig. 8F). This is consistent with a mechanism where targeting can enhance drug efficacy as FR-negative A549 cells show no measurable difference in cell viability responses toward fP-PCV_PTX_ and PCV_PTX_, for both incubation regimes (Fig. 8E,F).

The above data suggests that the optimized therapeutic window for any drug formulation must be derived separately for each cell type depending on the target receptor expression. The exposure time of a specific cancer cell to multivalent receptor-targeted therapeutics such as fP-PCV_PTX_ should be long enough to maximize effective receptor-mediated endocytosis but not so long as to have the uptake advantages being nullified by nonspecific processes. In this sense, it could be argued that our “click”-based *drop-in* strategy for the preparation of fP-PCV_PTX_ may provide a facile and modular strategy for tuning the density and morphology of targeting moieties to match the spectrum of biological receptor expression on the cell surface to arrive at the optimum therapeutic window.

### Summary

We have demonstrated a facile, modular strategy for the production of a targeted drug delivery system based on P4C6 vesicles. Our data show that these vesicles can effectively deliver a significant bolus of therapeutic agent to cancer cells. Once inside the cell, these vesicles can undergo triggered-release of the drug in cellular acidic microenvironments such as endosome. The incorporation of a terminal azide moiety into some of the amphiphilic molecules in the vesicles enables a modular, *drop-in* strategy for PCV functionalization, and allows for the conjugation of virtually any alkyne-modifiable targeting group onto PCVs without the loss of structural integrity or multivalent targeting capability. Coupled to the pronounced pH-sensitive release trigger of the P4C6 vesicles, the “clickable” PCV platform can facilitate the synthesis of a broad range of targeted therapeutics. As a proof of concept shown herein, folate-conjugated PCVs can be engineered to deliver drug payloads to specific receptor-positive tumour cells with high selectivity. The ability to engender stability, multivalent targeting capability, release trigger, and other functionalities into nanoscale drug delivery vehicles in a facile and modular fashion should make PCV a highly versatile platform that can significantly enhance the utility of nanoparticle delivery technology to tumours. Animal experiments will further be performed to evaluate the feasibility and safety of these strategies.

## Methods

### Materials

Unless otherwise noted, all reagents and materials were purchased from commercial sources and used as received. Dialysis bags were supplied by Spectrum Laboratories, Inc. (Rancho Dominguez, CA, cut off molecular weight of 3500 Da). Fluorescein isothiocyanate (FITC) and folic acid were purchased from Sigma-Aldrich Corporation (St Louis, MO, USA). Phosphate buffered saline (PBS), fetal bovine serum (FBS) were supplied by Life Technologies (Carlsbad, CA, USA). Paclitaxel (purity >99%) was purchased from Shanghai 21CEC Pharma (Shanghai, China). Ultrapure deionized water was obtained from a Millipore system (18.2 MΩ cm resistivity).

### Measurements

Fourier-transformed nuclear magnetic resonance (NMR) spectroscopy was performed on a Varian 400 MHz spectrometer in the Centre for Microscopy, Characterisation and Analysis (CMCA) facilities. Chemical shifts of ^1^H NMR spectra are reported in ppm against residual solvent resonance as the internal standard (CHCl_3_ = 7.27 ppm, CHD_2_COCD_3_ = 2.05 ppm, CHD_2_OD = 3.31 ppm).

Fourier-transformed infrared (FTIR) spectroscopy was performed on a Perkin Elmer Spectrum One FTIR spectrometer. KBr-pellets were prepared for FTIR measurements of alkyne-PEG-folate, azide-modified PCV, and click products. UV−vis absorption spectra were obtained on a CARY 50 Bio UV−vis spectrophotometer. Confocal Laser Scanning Microscopy (CLSM) studies were performed on a Leica TCS SP2 multiphoton confocal microscope. UV/Visible Detector used here is Waters 2998 Diode Array Detector. The diode array data was collected over the wavelengths 214–400 nm.

Chromatography was performed using a WATERS Prominence instrument (Waters MS Technologies, Manchester, UK) equipped with a 100 × 3.0 mm Symmetry C_18_, 3.5 μm column (Sunfire, Waters Corp, Milford, USA). The gradient elution mobile phase comprised of ACN (A) and 1 mM aqueous sodium acetate (B) mixture was delivered at a flow rate of 0.25 ml/min. The gradient was controlled as follows: 0–3.0 min, 20% A, 3.0–6.0 min, linear increase to 80% A, 6.0–9.0 min, 80% A, 9.0–12.0 min, linear decrease to 20% A. The column temperature was maintained at 40 °C and the autosampler was thermostatted at 4 °C. A 10.0 μL aliquot was injected into the LC/TOF MS system, and the total analytical run time was 12.0 min.

Mass spectrometry was performed on a Micromass LCT Premier system (Waters MS Technologies, Manchester, UK) operating in positive ion mode. The nebulization gas was set to 300 L/h at a temperature of 350 °C, the cone gas was 10 L/min and the source temperature was 80 °C. The capillary voltage and cone voltage were 3000 V and 60 V, respectively. The LCT-Premier was operated in the W optics mode with 12 000 resolution using the dynamic range extension (DRE). Data acquisition rate was set to 0.1 s, with 0.1 s interscan delay. All analyses were acquired using the lock spray to ensure accuracy and reproducibility; leucine-enkephalin was used as the lock mass (m/z 556.2771) at a concentration of 50 fmol/μL and flow rate of 5 μL/min. Data were collected in the centroid mode, with the lock spray frequency set at 5 s, and data were averaged over 10 scans.

Zeta potential and dynamic light scattering (DLS) measurements were performed on a Zetasizer Nano ZS (Marvern Instruments, Malvern, UK) with a He-Ne laser (633 nm) using a non-invasive backscatter method (detection at 173° scattering angle). Correlation data were fitted, using the method of cumulants, to the logarithm of the correlation function, yielding the diffusion coefficient, *D*. The hydrodynamic diameters (*D*_H_) of the PCVs were calculated using *D* and the Stokes−Einstein equation (*D*_H_ = *k*_B_*T*/3πη*D*, where *k*_B_ is the Boltzmann constant, *T* is the absolute temperature, and η is the solvent viscosity (η = 0.8872 cP for water). The polydispersity index (PDI) of vesicles (represented as 2*c/b*^2^, where *b* and *c* are first- and second-order coefficients, respectively, in a polynomial of a semilog correlation function) was calculated by the cumulants analysis. Size distribution of the vesicles was obtained by the non-negative least-squares (NNLS) analysis^32^. Unless noted otherwise, for DLS measurements all samples were dispersed in 10 mM HEPES solution (pH 7.4, 150 mM NaCl). The data reported represent an average of ten measurements with twenty scans each.

### Preparation of azide-Modified, Paclitaxel-loaded phosphonated Calix[4]arene Vesicles (PCV_PTX_)

Paclitaxel-loaded *p*-phosphonated calix[4]arene vesicles were prepared using a modified literature procedure^25^. To a cylindrical glass vial (15 mm × 45 mm) was added P4C6 (26.41 μmol), P3C6N3 (0.26 μmol, 1 mol % of the total P4C6), and paclitaxel (5.85 μmol) followed by chloroform (20 mL), affording a colorless solution. After vortexing (Heidolph Reax Top, John Morris Scientific Pty Ltd, Germany) 30 s, the solvent was removed by passing a stream of nitrogen over the solution while the vial was warmed in a 50 °C water bath. The resulting dry film was further dried under vacuum on a Schlenk line for one hour. Next, the dry lipid films were hydrated in 300 mM aqueous ammonium sulfate solution (500 μL) followed by vigorous probe sonication (2 min at 80% amplitude on Vibra cell Model VCX130, Sonics and Materials Inc, Australia) to form a dispersion of vesicles. The resulting PCV_PTX_ solution was purified by Sephadex G-50 (10 mL) gel-filtration chromatography that was pre-equilibrated with HEPES buffer (20 mM; [NaCl] = 150 mM, pH 7.4) and then was further used directly in the conjugation with azide-PEG-folate (see below).

The loading of the PTX was determined by breaking up the PTX-loaded vesicles using a modified literature procedure^25^. The PTX-loaded vesicles involved vortex-mixing 500 μl of each solution with 2.5 ml of ACN for 5 min, followed by centrifugation at 4820 g (5418 centrifuge, Eppendorf) for 10 min. The supernatant was transferred into glass autosampler vials and a 10 μl aliquot sample was injected into the LC/TOF MS system for analysis. Mean hydrodynamic diameter (*D*_H_) of 139 ± 16 nm was determined by DLS measurements (Fig. 2). The resulting alkyne-modified, PTX-loaded PCV (PCV_PTX_) can then be used directly in conjugation with azide-PEG-folate (see below).

The paclitaxel loading in the PCV was quantified by LC-TOF/MS using an adapted literature method^25^. Under set electrospray ionization conditions, paclitaxel was predominantly the protonated fragment [M+Na]^+^), and the MS parameters were optimized to maximize the response for the production of *m*/*z* 876.3224. The standard curve for paclitaxel was y = 255.21x + 62.18 (R^2 ^= 0.999). The percent drug loading efficiency (*D*_*L*_) was calculated from the mass of paclitaxel in PCV (*m*_*p*_) and the total mass of paclitaxel loaded PCV using equation 2; Encapsulation efficiency (E_E_) was calculated from the mass of paclitaxel in PCV (*m*_*p*_) and the total mass of feeding paclitaxel (*m*_*feeding paclitaxel*_) using equation 3.


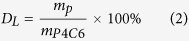



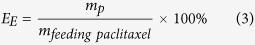


The loading efficiency (*D*_*L*_) and encapsulation efficiency (E_E_) were determined from three separately prepared vesicles suspensions, and was expressed as the mean ± standard deviation (Figure 3).

### PTX-Release Assay under Various pH Conditions

The release rate of paclitaxel from PCV_PTX_ was investigated using dialysis bags at pH 5.5 and 7.4. Here 10 mg PCV_PTX_ was placed into a dialysis bag (Spectrum Laboratories, Inc., Rancho Dominguez, CA, cut off molecular weight of 3500 Da) containing PBS (1000 ml) buffered to pH 5.5 or 7.4 with 1% Tween 80 and 0.5% FBS at 37.5 °C. At specific intervals the release medium which the dialysis bag was immersed in was sampled (5 mL) for analysis by the above mentioned LC/TOF MS method.

### Conjugation of Azide-PEG-folate Ligand to Alkyne-Modified PCV_PTX_ by Click Chemistry (Preparation of fP-PCV_PTX_)

To a solution containing the alkyne-modified PCV_PTX_ (570 μL of a 2.169 mM solution), azide-PEG folate ligand (24.72 μmol, 1 mol % of the total P4C6), CuSO_4_·5H_2_O (2 mM), and a freshly prepared aqueous solution of sodium ascorbate (1.2 mg in 300 μL of water, 6.18 μmol) was added and the reaction mixture was stirred at room temperature for 12 h. The resulting folate-conjugated PCV_PTX_ solution was purified by Sephadex G-50 (10 mL) gel-filtration chromatography which was pre-equilibrated with HEPES buffer (20 mM; [NaCl] = 150 mM, pH 7.4). To determine the final concentration of PTX in the as-prepared fP-PCV_PTX_, a small amount of this solution was first broken up by vortex-mixing 500 μl of each solution with 2.5 mL of ACN for 5 min, followed by centrifugation at 4820 g (5418 centrifuge, Eppendorf) for 10 min. The supernatant was transferred into glass autosampler vials and a 10 μl aliquot sample was injected into the LC/TOF MS system for analysis.

To determine the yield of the “click” reaction, the same conjugation described above was carried out with alkyne-modified empty PCVs (vide supra). This was necessary to avoid the overlapping UV−vis absorbances between PTX and folate in fP-PCV_PTX_. The yield of the conjugation with folate was determined by quantitative UV-vis spectroscopy based on the extinction coefficient (ϵ) of folic acid (λ_max_ = 291 nm, Fig. S2 in Supporting Information).

### Preparation of PCV_FITC_ and fP-PCV_FITC_

For detection by flow cytometry and confocal microscopy, 5 μmol FITC was incorporated in the place of PTX, as described above, to obtain PCV_FITC_ and fP-PCV_FITC_. PCV_FITC_ and fP-PCV_FITC_ are very stable (less than 5% FITC leaking from vesicles) in room temperature and neutral pH for 24 hours whereupon multiple dilutions applied (up to 1,000 times).

## Cell Culture Studies

### a. Medium

Trypsin solution (0.25%, containing EDTA) was purchased from Sigma-Aldrich (Castle Hill, AU). Ham’s F-12K (Kaighn’s) Medium, McCoy’s 5A (modified) Medium, Penicillin-Streptomycin, DAPI Nucleic Acid Stain, and phosphate-buffered saline (PBS, 1× without calcium and magnesium) solutions were purchased from Invitrogen (San Diego, CA).

### b. Cell Lines

Human ovarian adenocarcinoma (SKOV-3), known to overexpress the folate receptor was supplied from the American Type Culture Collection (ATCC). Human lung alveolar adenocarcinoma (A549) was used as the control cell line and was supplied from the ATCC. SKOV-3 cells were continuously cultured in folate-free McCoy’s 5A (modified) Medium supplemented with 10% (v/v) heat-inactivated fetal bovine serum (FBS) and 0.5% (v/v) Penicillin-Streptomycin solution at 37 °C in a humidified atmosphere containing 5% CO_2_. A549 cells were continuously cultured in Ham’s F-12K (Kaighn’s) Medium supplemented with 10% (v/v) FBS, 1% (v/v) Penicillin-Streptomycin solution at 37 °C in a humidified atmosphere containing 5% CO_2_ (Figs 6 and 7).

### Confocal Laser Scanning Microscopy (CLSM) and Flow Cytometry Study

SKOV-3 and A549 cells were planted in 12-well plate containing a sterilized glass coverslip (2 mL/well, 2.5 × 10^6 ^cells/mL) for 24 h at 37 °C and 5% CO_2_ before each experiment. Each cell was incubated with the appropriate FITC formulation in cell culture medium (500 μL) with or without free folate for 8 h at 37 °C and 5% CO_2_. After the removal of FITC-containing medium, the cell layers were washed with cold PBS (40 × 2 mL). Then 300 nM DAPI staining solution was added to the coverslip and made sure all the cells were completely covered. After incubation for 3 minutes, the samples were rinsed three times with PBS. Next, the coverslips were placed on slides (Livingstone Ltd, NSW, AU) coated with PBS (50 μL). CLSM images were observed with inverted confocal laser scanning microscope (Leica TCS SP2 multiphoton confocal microscope) with excitation at 488 nm. A NA1.4 oil immersion objective was used. The rest of the cells were digested using 0.25% Trypsin. After centrifugation, the resulting pellet was redispersed in pH 7.4 PBS for flow cytometry analysation (Figure 8).

### Cytotoxicity Assays

The cells were seeded in 6-well plates (500 μL/well) with a concentration of 2.5 × 10^6 ^cells/mL in folate-free medium and were incubated to grow for 24 h. The media in the wells were replaced with the preprepared growth media containing the appropriate drug formulation (200 μL of solution at the appropriate paclitaxel concentrations (0.01–900 μM). For exposure regime 1, the drug-treated cells were then further incubated for 4 h in a humidified atmosphere containing 5% CO_2_ at 37 °C, after which the drug-containing media were removed by aspiration. The remaining cell layers were washed with PBS buffer (2 × 250 μL) followed by replacement with fresh growth media (200 μL). The plates were then maintained in the incubator at 37 °C for a further 68 h. For exposure regime 2, the drug-treated cells were incubated for 72 h in a humidified atmosphere containing 5% CO_2_ at 37 °C, after which the cells were washed with PBS buffer (2 × 250 μL).

The total cells from cultures were differentially stained with Dead Cell Apoptosis Kit (Life Technologies, San Diego, CA) which contained two fluorescence dyes (Annexin V FITC and PI), allowing assessment of the viable, dead, and apoptotic cells. The number of viable cells was counted by a BD FACSCalibur flow cytometer and was plotted against the total PTX concentration. The data reported represent an average of three measurements from different batches. The dose-response curves shown in Fig. 8 were obtained by sigmoidal logistic fitting using Origin 8.5 and the half-maximal inhibitory concentration (IC_50_) values were determined on the basis of the fitted data.

## Additional Information

**How to cite this article**: Mo, J. *et al.* Paclitaxel-loaded phosphonated calixarene nanovesicles as a modular drug delivery platform. *Sci. Rep.* **6**, 23489; doi: 10.1038/srep23489 (2016).

## Supplementary Material

Supplementary Information

## Figures and Tables

**Table 1:** Half-maximal inhibitory concentration (IC_50_) (μM, mean ± SD, *n* = 3) values of free PTX, PCV_PTX_, and fP-PCV_PTX_ against SKOV-3 and A549 cell lines.

Short Incubation Regime (4 h pulsed exposure)					Long Incubation Regime (72 h continuous exposure)			
Cell lines	Free PXT	PCV_PTX_	fP-PCV_PTX_	fP-PCV_PTX_ with free folate	Free PXT	PCV_PTX_	fP-PCV_PTX_	fP-PCV_PTX_ with free folate
SKOV-3	3.79 ± 0.31	8.68 ± 0.88	2.42 ± 0.29	5.87 ± 0.56	0.30 ± 0.03	0.94 ± 0.11	0.43 ± 0.04	0.62 ± 0.05
A549	2.25 ± 0.19	8.73 ± 0.91	7.88 ± 0.82	6.42 ± 0.55	0.16 ± 0.02	0.49 ± 0.04	0.40 ± 0.05	0.47 ± 0.04

## References

[b1] Torchilin V. P. Recent advances with liposomes as pharmaceutical carriers. Nat Rev Drug Discov 4, 145–160, doi: 10.1038/nrd1632 (2005). 15688077

[b2] Jiang L. *et al.* Anti-Cancer Efficacy of Paclitaxel Loaded in pH Triggered Liposomes. J Biomed Nanotechnol 12, 79–90, doi: 10.1166/jbn.2016.2123 (2016). 27301174

[b3] Jiang L. *et al.* Overcoming drug-resistant lung cancer by paclitaxel loaded dual-functional liposomes with mitochondria targeting and pH-response. Biomaterials 52, 126–139, doi: 10.1016/j.biomaterials.2015.02.004 (2015). 25818419

[b4] Bae Y., Jang W. D., Nishiyama N., Fukushima S. & Kataoka K. Multifunctional polymeric micelles with folate-mediated cancer cell targeting and pH-triggered drug releasing properties for active intracellular drug delivery. Mol Biosyst 1, 242–250, doi: 10.1039/b500266d (2005). 16880988

[b5] Wang Y. *et al.* Polysorbate 80 coated poly (varepsilon-caprolactone)-poly (ethylene glycol)-poly (varepsilon-caprolactone) micelles for paclitaxel delivery. Int J Pharm 434, 1–8, doi: 10.1016/j.ijpharm.2012.05.015 (2012). 22609127

[b6] Gao X. *et al.* Combined Delivery and Anti-Cancer Activity of Paclitaxel and Curcumin Using Polymeric Micelles. J Biomed Nanotechnol 11, 578–589, doi: 10.1166/jbn.2015.1964 (2015). 26310065

[b7] Rui Y., Wang S., Low P. S. & Thompson D. H. Diplasmenylcholine–Folate Liposomes: An Efficient Vehicle for Intracellular Drug Delivery. J Am Chem Soc 120, 11213–11218, doi: 10.1021/ja9742949 (1998).

[b8] Zhao X. B. & Lee R. J. Tumor-selective targeted delivery of genes and antisense oligodeoxyribonucleotides via the folate receptor. Adv Drug Deliver Rev 56, 1193–1204, doi: 10.1016/j.addr.2004.01.005 (2004). 15094215

[b9] Lu Y. & Low P. S. Folate-mediated delivery of macromolecular anticancer therapeutic agents. Adv Drug Deliver Rev 54, 675–693, doi: 10.1016/S0169-409X(02)00042-X (2002). 12204598

[b10] Yoo H. S. & Park T. G. Folate-receptor-targeted delivery of doxorubicin nano-aggregates stabilized by doxorubicin-PEG-folate conjugate. J Control Release 100, 247–256, doi: 10.1016/j.jconrel.2004.08.017 (2004). 15544872

[b11] Muller C. & Schibli R. Folic acid conjugates for nuclear imaging of folate receptor-positive cancer. J Nucl Med 52, 1–4, doi: 10.2967/jnumed.110.076018 (2011). 21149477

[b12] Tran P. H., Tran T. T. & Lee B. J. Biodistribution and pharmacokinetics in rats and antitumor effect in various types of tumor-bearing mice of novel self-assembled gelatin-oleic acid nanoparticles containing paclitaxel. J Biomed Nanotechnol 10, 154–165, doi: 10.1166/jbn.2014.1660 (2014). 24724507

[b13] Martin A. D. *et al.* Synthesis and Toxicology of p-Phosphonic Acid Calixarenes and O-Alkylated Analogues as Potential Calixarene-Based Phospholipids. ChemPlusChem 77, 308–313, doi: 10.1002/cplu.201100081 (2012).

[b14] James E. *et al.* Antioxidant phospholipid calix[4]arene mimics as micellular delivery systems. Org Biomol Chem 11, 6108–6112, doi: 10.1039/C3OB41178H (2013). 23921718

[b15] Eggers P. K. *et al.* Composite fluorescent vesicles based on ionic and cationic amphiphilic calix[4]arenes. Rsc Adv 2, 6250–6257, doi: 10.1039/C2ra20491f (2012).

[b16] Mo J. *et al.* Shear induced carboplatin binding within the cavity of a phospholipid mimic for increased anticancer efficacy. Sci. Rep. 5, doi: 10.1038/srep10414 (2015). PMC5386247 26000441

[b17] Gupta N., Hatoum H. & Dy G. K. First line treatment of advanced non-small-cell lung cancer - specific focus on albumin bound paclitaxel. Int J Nanomed 9, 209–221, doi: 10.2147/IJN.S41770 (2014). PMC3875520 24399877

[b18] Wang C. *et al.* Synthesis, characterization, and application of amino-terminated poly(ethylene glycol)-block-poly(epsilon-caprolactone) copolymer for paclitaxel. J Nanosci Nanotechno 13, 68–76, doi: 10.1166/jnn.2013.6695 (2013). 23646699

[b19] Wang C. *et al.* Characterization, pharmacokinetics and disposition of novel nanoscale preparations of paclitaxel. Int J Pharm 414, 251–259, doi: 10.1016/j.ijpharm.2011.05.014 (2011). 21596124

[b20] Guo J. *et al.* Poly-, ß-Polyasparthydrazide-Based Nanogels for Potential Oral Delivery of Paclitaxel: *In vitro* and *In vivo* Properties. J Biomed Nanotechnol 11, 2231–2242, doi: 10.1166/jbn.2015.2118 (2015). 26510316

[b21] Ye L. *et al.* Antitumor effect and toxicity of Lipusu in rat ovarian cancer xenografts. Food Chem Toxicol 52, 200–206, doi: 10.1016/j.fct.2012.11.004 (2013). 23149094

[b22] Xu X. *et al.* Clinical comparison between paclitaxel liposome (Lipusu(R)) and paclitaxel for treatment of patients with metastatic gastric cancer. Asian Pac J Cancer P 14, 2591–2594 (2013). 10.7314/apjcp.2013.14.4.2591 23725180

[b23] Lim R. K. & Lin Q. Bioorthogonal chemistry: recent progress and future directions. Chem Commun (Camb) 46, 1589–1600, doi: 10.1039/b925931g (2010). 20177591 PMC2914230

[b24] Li X., Guo J., Asong J., Wolfert M. A. & Boons G.-J. Multifunctional Surface Modification of Gold-stabilized Nanoparticles by Bioorthogonal Reactions. J Am Chem Soc 133, 11147–11153, doi: 10.1021/ja2012164 (2011). 21678979 PMC3153077

[b25] Mo J. X., Eggers P. K., Raston C. L. & Lim L. Y. Development and validation of a LC/TOF MS method for the determination of carboplatin and paclitaxel in nanovesicles. Anal Bioanal Chem 406, 2659–2667, doi: 10.1007/s00216-014-7684-0 (2014). 24573580

[b26] Tannock I. F. & Rotin D. Acid pH in tumors and its potential for therapeutic exploitation. Cancer Res 49, 4373–4384 (1989). 2545340

[b27] Kumler W. D. & Eiler J. J. The Acid Strength of Mono and Diesters of Phosphoric Acid. The n-Alkyl Esters from Methyl to Butyl, the Esters of Biological Importance, and the Natural Guanidine Phosphoric Acids. J Am Chem Soc 65, 2355–2361, doi: 10.1021/ja01252a028 (1943).

[b28] Leamon C. P., Cooper S. R. & Hardee G. E. Folate-liposome-mediated antisense oligodeoxynucleotide targeting to cancer cells: evaluation *in vitro* and *in vivo*. Bioconjugate Chem 14, 738–747, doi: 10.1021/bc020089t (2003). 12862426

[b29] Saul J. M., Annapragada A., Natarajan J. V. & Bellamkonda R. V. Controlled targeting of liposomal doxorubicin via the folate receptor *in vitro*. J Control Release 92, 49–67, doi: 10.1016/S0168-3659(03)00295-5 (2003). 14499185

[b30] Kramer R. A., Zakher J. & Kim G. Role of the glutathione redox cycle in acquired and *de novo* multidrug resistance. Science 241, 694–697 (1988). 3399900 10.1126/science.3399900

[b31] Pizzato M. *et al.* Evidence for nonspecific adsorption of targeted retrovirus vector particles to cells. Gene Ther 8, 1088–1096, doi: 10.1038/sj.gt.3301494 (2001). 11526456

[b32] Schätzel K., Neumann W.-G., Müller J. & Materzok B. Optical tracking of single Brownian particles. Appl. Opt. 31, 770–778, doi: 10.1364/ao.31.000770 (1992). 20720683

