# Supplementary material for: Paclitaxel-loaded phosphonated calixarene nanovesicles as a modular drug delivery platform
Source: Sci Rep. 2016 Mar 24;6:23489. doi: 10.1038/srep23489 (PMC4806332; doi:10.1038/srep23489)
Supplement: Supplementary Information [file srep23489-s1.doc]

**Supporting Information**

**Paclitaxel-loaded phosphonated calixarene nanovesicles as a modular drug delivery platform**

*Jingxin Mo, Paul K. Eggers, Zhi-xiang Yuan, Colin L. Raston and*

*Lee Yong Lim*


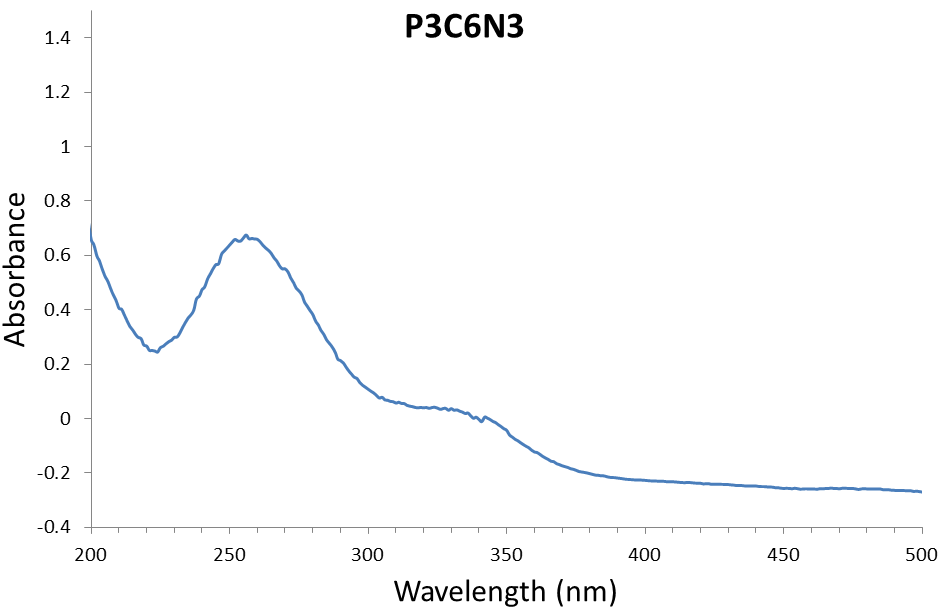

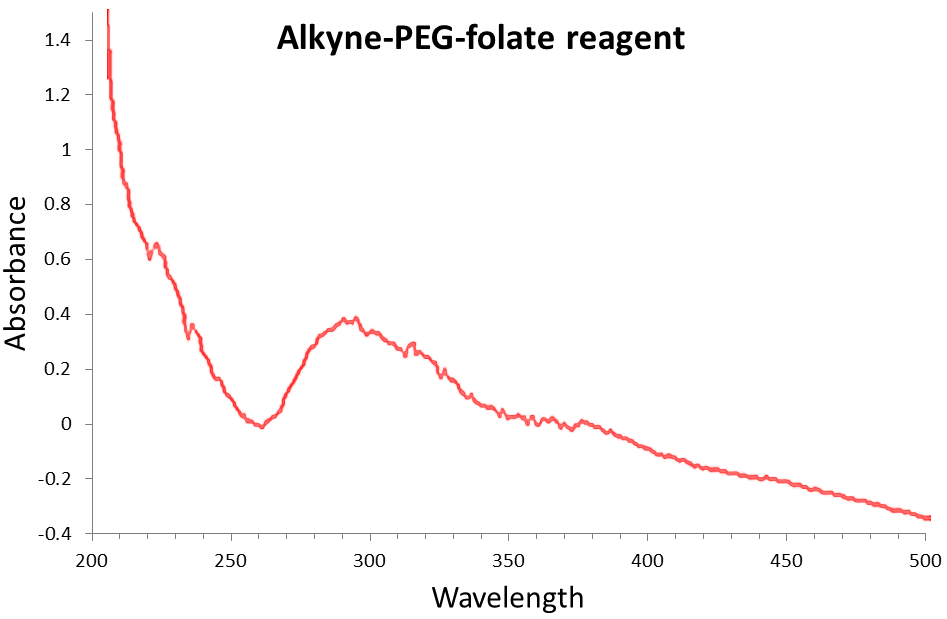


**B**

**A**


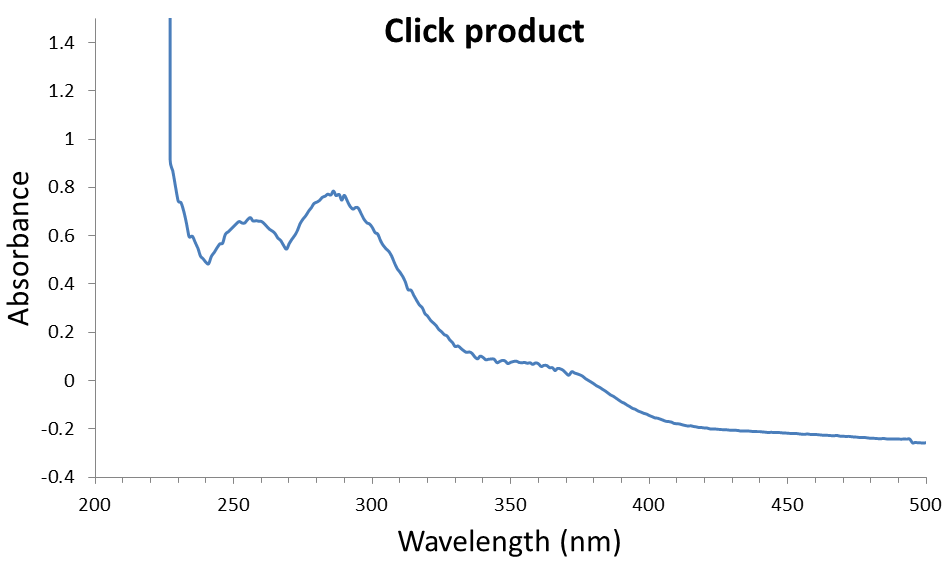


**C**

**Figure S1.** UV-vis spectra of click reactants P3C6N3 (A), alkyne-PEG-FA (B), and click products (C), after purification.

**
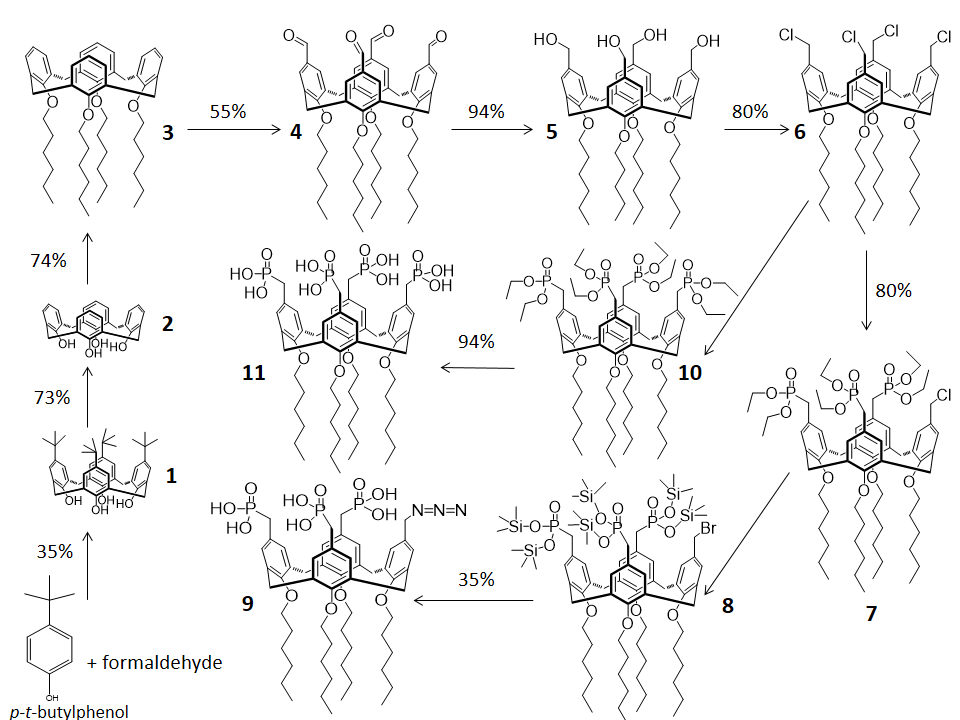
**

**Scheme 3.** Synthesis of P4C6 and P3C6N3.

**1. Synthesis of 5,11,17,23-tetrakis(1,1-dimethylethyl)-25,26,27,28-tetra-hydroxycalix[4]arene (1).** A 2 L three-necked round bottom flask with a thermometer attached was purged with argon. *P*-*t*-butylphenol (170.8 g, 1.137 mol), formaldehyde (>25 % wt. water solution, 108 mL), and NaOH (9.78 M, 2.3 mL) were placed in the flask and heated to 120 °C. Vapour was allowed to escape the flask under argon and a thick, viscous homogenous foam formed whereupon the heating was switched off but was allowed to stir. Recycled phenyl ether (1.3 L, distilled *in vacuo*) and toluene (80 mL) was added, and the heating mantle was set to 260 °C. The azeotropic removal of water was allowed to occur (internal temperature 180-190 °C) before the light brown mixture was refluxed overnight (8 h). Heating was stopped and the dark brown reaction left to cool to 60 °C. Ethyl acetate (1 L) was added, and the mixture was cooled to room temperature and stirred for 4 hours. The brown solid was vacuum filtered (brown tinted mother liquor kept for recycling) and triturated with acetic acid (130 ml). The light brown solid was vacuum filtered and washed with ethyl acetate (4×30 ml). The resulting solid was dissolved in toluene (1.8 L) at 100 °C, cooled slowly to room temperature and vacuum filtered. The resulting glistening, flaky white crystals of **1** was air dried (65.2 g, 35 %).

^1^H NMR (CDCl_3_, 500 MHz) δ: 1.21 (s, 36 H), 3.50, 4.22 (2d, *J*= 13.5 Hz, 2×4 H), 7.04 (s, 8 H), 10.32 (s, 4 H) ppm.

**2. Synthesis of 25,26,27,28-tetra-hydroxycalix[4]arene (2).** A 2L three-necked round bottom flask was dried in the oven, attached to an aspirator in a 2 M NaOH solution, to trap evolved HCl and purged with argon. The flask was loaded with **1** (31.2 g, 0.0481 mol), dissolved in toluene (200 mL) and phenol (5.3 g) was added. The mixture was stirred for 10 minutes. Aluminium trichloride (AlCl_3_, 60 g) was carefully weighed in the fume hood and added to the mixture. The mixture then turned murky, deep red and sticky, and was left to stir overnight. Dichloromethane (500 mL) was added and the mixture was stirred for 2 hours.= whereupon the solution was cooled in an ice bath and then cold water (300 mL) was added. The organic layer was extracted, washed with HCl (3×200 mL) and water (3×200 mL) and dried over anhydrous sodium sulfate. The organic extract was distilled *in vacuo* and the product recrystallised from diethyl ether to give a pale yellow solid, **2** (14.9 g, 73 %).

^1^H NMR (CDCl_3_, 500 MHz) δ: 3.15, 4.46 (2d, *J*= 13.5 Hz, 2×4 H), 6.55 (m, 12 H), 10.55 (s, 4 H) ppm.

**3. Synthesis of 25,26,27,28-tetra-hexyloxycalix[4]arene (3).** Sodium hydride (NaH, 8.2 g) was carefully triturated in hexane in a 250 ml Erlenmeyer and left to settle under argon for an hour. **2** (6.02 g, 0.0142 mol) was weighed out in a 500 mL round bottom flask and placed on the receptacle of the distillation apparatus for dimethylformamide (DMF). DMF (100 mL) was distilled and the mixture was left to stir under argon to dissolve **2**. An ethanol squirt bottle was readied in case of NaH spill, and the hexane was carefully decanted off the NaH and dry DMF (100 mL) added to the Erlenmeyer. The NaH/DMF solution was carefully added to the stirring solution of **2** and left to stir for 20 minutes under argon. 1-bromohexane (16.2 mL, 0.115 mol) was pipetted into the stirring solution and left to stir under the argon for an hour. The flask was then attached to a condenser, the reaction heated to 100 °C and then refluxed overnight. The reaction flask was then cooled in an ice bath, water (50 mL) was slowly added, followed by drop wise addition of hydrochloric acid (HCl, 2 M, 200 mL) and left to stir for 30 minutes. The product was extracted with dichloromethane (DCM, 200 mL), and the foamy organic layer was washed with HCl (2 M, 200 mL) and brine/HCl (50:50, 2 M, 3×200 mL). The organic extract is distilled *in vacuo* and resulted in a pale yellow oil of **3** (8.06 g, 74 %).

^1^H NMR (CDCl_3_, 500 MHz) δ: 0.81 (t, *J*=7.15 Hz, 12 H), 1.12 (m, 32 H), 1.87 (m, 8 H), 3.15, 4.46 (2 d, *J*= 13.5 Hz, 2×4 H), 6.61 (t, *J*=7.45 Hz, 8 H) ppm.

^13^C NMR (CDCl_3_, 128.5 MHz) δ: 14.2 (CH_3_), 22.0–31.0 (4×CH_2_), 32.4 (CH_2_), 134.5 (CH), 135.7 (C), 140.2 (C), 165.2 (C) ppm.

**4. Synthesis of 5,11,17,23-tetraformyl-25,26,27,28-tetra-hexyloxycalix[4]arene** **(4)., 3** (8.06 g, 0.01045 mol) and hexamethylenetetramine (69.0 g, 0.492 mol) was loaded in a 500 mL round bottom flask with a stirrer bar, heated to 50 °C and dried *in vacuo* for 4 hours. Trifluoroacetic acid (300 mL) was added to the solid mixture and stirred. The reaction was heated to 100 °C and refluxed overnight under argon. The reaction mixture turned from red to green. The reaction mixture was cooled, added to a vigorously stirring solution of HCl (2 M, 1.2 L) and DCM (600 mL), and stirred overnight. The organic extract was washed with HCl:brine (3:2, 2 M, 6×500 mL) to remove degraded and excess hexamethylenetetramine that will interfere with the next step of purification. The organic extract was distilled *in vacuo* and purified via column chromatography (mobile phase - ethyl acetate:chloroform = 5:1, *R_f_* = 0.82) to yield pale yellow solids of **4** (5.04 g, 55 %).

^1^H NMR (CDCl_3_, 500 MHz) δ: 0.81 (t, *J*=7.15 Hz, 12 H), 1.12 (m, 32 H), 1.87 (m, 8 H), 3.22, 4.20 (2 d, *J*= 13.5 Hz, 2×4 H), 7.02 (s, 8 H), 9.21 (s, 8 H) ppm.

^13^C NMR (CDCl_3_, 128.5 MHz) δ: 13.9 (CH_3_), 22.0–30.7 (4×CH_2_), 31.8 (CH_2_), 130.0 (CH), 131.2 (C), 135.5 (C), 161.7 (C), 190.9 (CH) ppm.

**5. Synthesis of 5,11,17,23-tetramethylhydroxy-25,26,27,28-tetra-hexyloxycalix[4]arene** (**5**)**. 4** (2.54 g, 2.91 mmol) was dissolved in tetrahydrofuran (THF, 10 mL) in a 500 mL round bottom flask and stirred. Ethanol (EtOH, 60 mL) was added and the mixture turned cloudy. Sodium borohydride (NaBH_4_, 8.2 g, 0.22 mol) was added to the stirring mixture and left to stir overnight. The solution was distilled *in vacuo* followed by the addition of DCM and stirred (150 mL). The solution was placed in an ice bath and HCl (2 M, 100 mL) was added until the solution turned basic. The organic extract was washed with HCl (2 M, 4×400 mL) and saturated sodium carbonate solution (Na_2_CO_3 (_*_sat_*), 4×400 mL). The organic extract was distilled *in vacuo* and white solids of **5** (2.40 g, 94 %)

^1^H NMR (CDCl_3_, 500 MHz) δ: 0.84 (t, *J*=7.15 Hz, 12 H), 1.25 (m, 32 H), 2.25 (m, 8 H), 3.145, 4.55 (2 d, *J*= 13.25 Hz, 2×4 H), 4.01 (s, 8 H), 6.85 (s, 8 H) ppm.

^13^C NMR (CDCl_3_, 128.5 MHz) δ: 15.5 (CH_3_), 21.0–31.0 (4×CH_2_), 30.8 (CH_2_), 70.4 (CH_2_), 110.8 (CH), 115.6 (C), 120.5 (C), 145.2 (C) ppm.

**6. Synthesis of 5,11,17,23-tetrachloromethyl-25,26,27,28-tetra-hexyloxycalix[4]arene** (**6**)**. 5** (149.6 mg, 0.1698 mmol) was accurately weighed and loaded onto a dry 50 mL round bottom flask. Thionyl chloride (10 mL) was added carefully and stirred overnight with a drying tube attached. The thionyl chloride was carefully distilled *in vacuo* and yellow oily solids of **6** (130.4 mg, 80 %) was obtained.

^1^H NMR (CDCl_3_, 500 MHz) δ: 0.92 (t, *J*=7.15 Hz, 12 H), 1.2 (m, 32 H), 2.0 (m, 8 H), 3.17, 4.44 (2 d, *J*= 13.5 Hz, 2×4 H), 3.95 (t, *J*=7.435 Hz, 8 H), 4.40 (s, 8 H) ppm.

^13^C NMR (CDCl_3_, 128.5 MHz) δ: 14.4 (CH_3_), 22.2-31.0 (4×CH_2_), 32.8 (CH_2_), 46.2 (CH_2_), 75.8 (CH_2_), 129.4 (CH), 131.5 (C), 136.4 (C), 160.2 (C) ppm.

**7. Synthesis of 5,11,17,23-tetra-[tri-(diethyl)phosphonomethyl]chloromethyl-25,26,27,28-tetra-hexyloxycalix[4]arene** (**7**)**.** Dry dioxane (25 mL) was used to dissolve **6** (130.4 mg, 0.1365 mmol) in a glass pressure tube filled with triethyl phosphite and sealed. The pressure tube was heated to 200 °C and stirred for 3 days. The solution was distilled *in vacuo* and resulting brown oil of **7** (147.8 mg, 79.5 %) was obtained.

^1^H NMR (CDCl_3_, 500 MHz) δ: 0.75 (t, *J*=6.85 Hz, 12 H), 1.25 (m, 32 H), 1.83 (m, 8 H), 2.90 (d, *J*=21.2 Hz, 8 H), 3.01, 4.25 (2d, *J*= 13.5 Hz, 2×4 H), 3.69 (t, *J*=7.25 Hz, 8 H), 6.44 (s, 8 H) ppm.

^13^C NMR (CDCl_3_, 128.5 MHz) δ:13.8 (CH_3_), 16.0 (d,CH_3_,7.0Hz), 22.4-32.0 (4×CH_2_), 33.4 (d,CH_2_,138 Hz), 66.4 (CH_2_), 74.8 (CH_2_), 14.5 (C), 130.8 (CH), 135.4 (C), 155.8 (C) ppm.

**8. Synthesis of 5,11,17,23-tetra-tri-[di-(trimethylsilyl)]phosphonomethyl-bromomethyl-25,26,27,28-tetra-hexyloxycalix[4]arene (8). 7** (50.4 mg, 0.0370 mmol) was stirred in bromotrimethylsilane (1.2 ml) in a 50 mL round bottom flask overnight under argon. Acetonitrile (5 mL) was added and distilled *in vacuo*. The resulting yellow oil of **8** was used immediately for the attachment of azide in the next step.

**9. Synthesis of 5,11,17,23-tetra-(triphosphonomethyl)azidomethyl-25,26,27,28-tetra-hexyloxycalix[4]arene (9).** Sodium azide (0.5 g) was added to the 50 mL round bottom flask containing **8**, and positioned at the receptacle of a distillation apparatus with DMF. The flask is filled with dry DMF (20 mL) and the reaction is stirred and refluxed at 100°C under argon overnight. The mixture was placed in the freezer for 2 hours and filtered. The mother liquor was distilled *in vacuo* to result solids of **9** (14.2 mg, 35 %).

^13^C NMR (CDCl_3_, 128.5 MHz) δ: 14.01 (CH_3_), 29.0-31.0 (4×CH_2_), 32.1 (CH_2_), 54.6 (m, CH_3_), 55.7 (m, CH_2_), 69.8 (m, CH_2_), 76.3 (m, CH_2_), 122.9 (m, C), 131.2 (m, CH), 134.7 (m, CH), 135.9 (m, C), 159.7 (m, C) ppm.

**10. Synthesis of 5,11,17,23-tetra-diethylphosphonomethyl-25,26,27,28-tetra-hexyloxycalix[4]arene (10).**  Dry 5,11,17,23-tetramethylchloride-25,26,27,28-tetra-hexyloxycalix[4]arene (150 mg, 0.162 mmol) was added with stirring triethylphosphite (15 mL) and the mixture was then refluxed for 16 h. The solution was cooled to room temperature and the triethylphosphite removed under reduced pressure. The raw product was purified by column chromatography (silica gel 60; chloroform : methanol, 10 : 1) to give a white solid for next reaction.

**11. Synthesis** **of 5,11,17,23-tetra-phosphonomethyl-25,26,27,28-tetra-****hexyloxycalix[4]arene (11).** Under an inert gas atmosphere dry 5,11,17,23-tetra-methylchloride-25,26,27,28-tetra-tetrahexyloxycalix[4]arene (**10,** 159.1 mg, 0.117 mmol) was added to bromotrimethylsilane (3 mL) and stirred at room temperature for 5 h. The bromotrimethylsilane was removed under reduced pressure. The resulting solid was dissolved in water (5 mL) and stirred for 2 h. The resultant white solid that formed was collected via vacuum filtration (125.0 mg, 94 %).

^1^H NMR (MeOD/CDCl_3_, 600.1 MHz) d: 0.79 (t, *^3^J* = 6.90 Hz, 1 2H), 1.25 (m, 32 H), 1.87 (m, 8 H), 2.82 (m, 8 H), 3.03, 4.30 (2 d, ^2^*J* = 12.7 Hz, 2×4 H), 3.72 (t, ^3^*J* = 7.55 Hz, 8 H), 6.67 (s, 8 H).

^13^C NMR (MeOD/CDCl_3_, 150.9 MHz) d: 13.9 (CH_3_), 22.6 (CH_2_), 29.3 (CH_2_), 29.7 (CH_2_), 29.9 (CH_2_), 31.8 (CH_2_), 33.5 (CH_2_), 75.4 (CH_2_), 125.5 (C), 129.6 (CH), 134.7 (C), 154.9 (C).

TOF MS ES^-^ (*m/z*): Expected for C_56_H_82_O_16_P_4_^2-^, 1134.4710. Found: 1134.4682.
